# Supplementary material for: Partner choice and cooperation in social dilemmas can increase resource inequality
Source: Nat Commun. 2023 Oct 13;14:6432. doi: 10.1038/s41467-023-42128-2 (PMC10575984; doi:10.1038/s41467-023-42128-2)
Supplement: Supplementary file 1 — Supplementary Information [file 41467_2023_42128_MOESM1_ESM.pdf]

Supplementary Information

Partner choice and cooperation in social dilemmas can increase resource inequality

Mirre Stallen, Luuk Snijder, Jörg Gross, Leon P. Hilbert, and Carsten K.W. De Dreu

Correspondence to: l.snijder@fsw.leidenuniv.nl

This file includes:

Supplementary Note 1
Supplementary Note 2
Supplementary Fig. 1
Supplementary Note 3 (Supplementary Tables 1 to 14)

Supplementary Note 1

Between platform analyses

As participants were recruited via Leiden University and via Prolific, we examined, for each multilevel (logistic) model reported in the manuscript and in the Supplementary Information, whether participant pool mattered for our main results and conclusions. To this end, we computed additional models with participant pool as a covariate, and with interaction terms between participant pool and our experimental manipulations. In total and across dependent variables, this resulted in 59 possible interactions including participant pool. Because we had no a priori hypotheses, we applied Bonferroni-correction to avoid Type I errors (i.e., with $p = 0.05$ and 59 tests, interaction terms with $p < 0.0009$ are considered informative). Four interaction terms were significant, indicating differences in behaviour between our samples. First, participants who were assigned an LL type were paired more often to each other in the partner choice condition in the Prolific compared to the Leiden University pool ($z = 4.71$, $b_{\text{LL} \times \text{platform}} = 1.89$, $p < 0.0001$, 95% CI [1.26, 2.55]; $z = -3.43$, $b_{\text{LL} \times \text{condition} \times \text{platform}} = -1.90$, $p = 0.0006$, 95% CI [-2.63, -1.12]; base model in Supplementary Table 1). Second, cooperation in the partner choice condition increased over time in the 5 groups recruited via Prolific and decreased over time in the 16 groups recruited via Leiden University ($t(7724) = 7.04$, $b_{\text{round} \times \text{platform}} = 0.89$, $p < 0.0001$, 95% CI [0.64,1.14]; base model in Supplementary Table 8). Likewise, cooperation of one's partner increased over time in the 5 groups recruited via Prolific, and decreased over time in the 16 groups recruited via Leiden University ($t(2898) = 5.59$, $b_{\text{round} \times \text{platform}} = 1.05$, $p < 0.0001$, 95% CI [0.68,1.42]; base model in Supplementary Table 10).

Supplementary Note 2

Additional information on our pre-registered pilot study

The first study as reported in the pre-registration was conducted in a seminar setting with 70 participants to test the clarity of experimental instructions and the reliability of the computer interfaces. Groups played 16 rounds (rather than 24, as in the main experiment). Two out of nine groups had less than 8 actual participants, and missing participants were replaced by preprogrammed bots. Because of this, and the low number of groups without bots, formal data analysis was deemed uninformative and not performed.

Supplementary Figure

Supplementary Fig. 1 shows the relative cooperation towards partner types in the partner choice condition, depicted separately for participants' own type. Participants who were assigned an HH type cooperated less when being paired with participants assigned an LH or LL type than when being paired with participants assigned an HH or HL type. However, cooperation of participants who were assigned an LL type did not depend on their partner's assigned type.

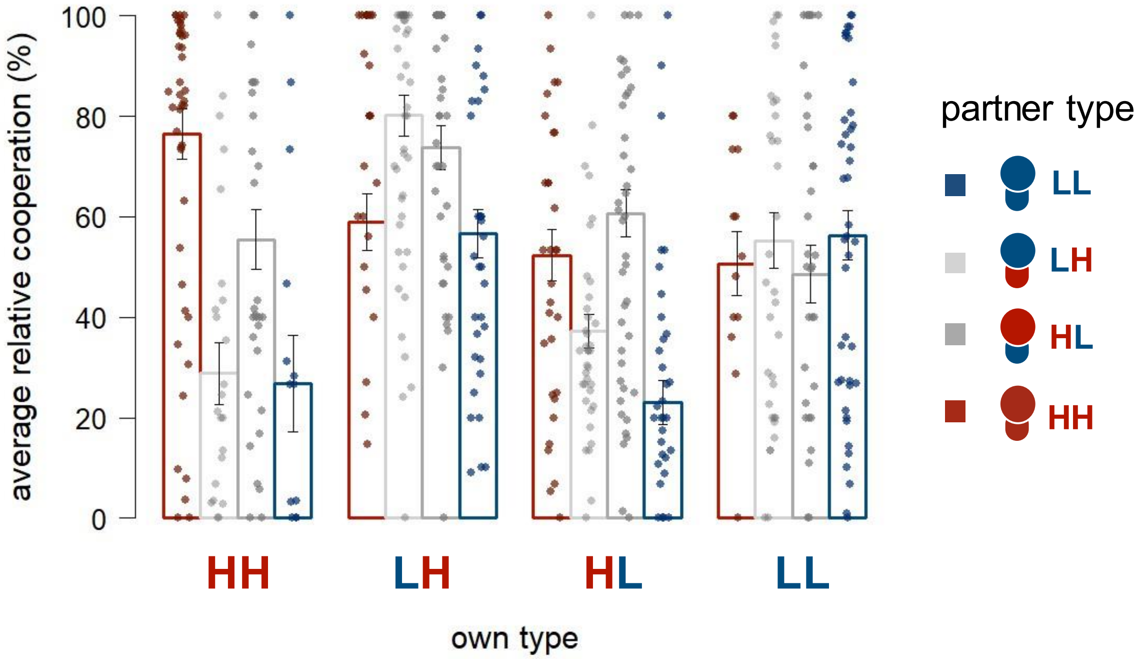

Supplementary Fig. 1. Cooperation towards each partner type, for each participant type. Cooperation differed between types in the partner choice condition (n = 21 groups), so that participants who were assigned a high endowment (i.e., HH and HL types) cooperated less with participants who were assigned a low endowment (i.e., LH and LL types). Error bars indicate the standard error of the mean. Dots show averages per participant.

Supplementary Note 3

Supplementary Tables 1-14

If multiple contrasts were analysed within the same model, we corrected for multiple testing using a Bonferroni correction.

Segregation under Partner Choice

All multilevel (logistic) regression models reported under *Segregation under Partner Choice* included random intercepts for participants nested within their group to account for violations of independence, since participants made repeated decisions and were part of a group in which they potentially influenced each other's decisions over time.

Segregation

We fit a multilevel logistic regression model to test how often types were paired in the partner choice and assigned partner condition. The dependent variable in the model was a dummy variable coding whether similar types were paired (1), or not (0). The fixed effect consisted of participants' assigned type, the condition, and the interaction between type and condition.

In the assigned partner condition, pairs were uniformly distributed by design, meaning that participants interacted an equal number of times with each possible type. This pattern did not evolve in the partner choice condition. Here, we observed mostly same type pairs (multilevel logistic model, $z = -7.56$, $b_{\text{condition}} = -1.68$, $p < .001$, 95% CI [-2.18, -1.19], Supplementary Table 1). Furthermore, in the partner choice condition, HH and LL types were paired more often with their own type than HL and LH types were paired with their own type (multilevel logistic model, $z = 8.74$, $b_{\text{type}} = 2.15$, $p < .001$, 95% CI [1.60, 2.70], Supplementary Table 1).

Supplementary Table 1. Mixed effects logistic regression modelling how often similar types were paired per condition.

		Estimate	SE	z	p	95% CI
**Model**	intercept	1.31	0.19	6.76	< 0.001	[0.93, 1.70]
	HL type	-1.30	0.18	-7.40	< 0.001	[-1.66, -0.96]
	LH type	-1.30	0.18	-7.37	< 0.001	[-1.66, -0.96]
	LL type	-0.45	0.18	-2.57	0.010	[-0.81, -0.11]
	assigned partner condition	-2.45	0.27	-9.05	< 0.001	[-2.99, -1.92]
	HL type × assigned partner condition	1.30	0.24	5.34	< 0.001	[0.83, 1.79]
	LH type × assigned partner condition	1.30	0.24	5.32	< 0.001	[0.82, 1.79]
	LL type × assigned partner condition	0.45	0.24	1.86	0.063	[-0.03, 0.94]
**Contrasts**[a]	contrast 1	-1.68	0.22	-7.56	< 0.001	[-2.18, -1.19]
	contrast 2	2.15	0.25	8.74	< 0.001	[1.60, 2.70]

[a] contrast 1 tests (two-sided) whether the distribution of pairs differed between the partner choice and assigned partner condition ($p < .001$), contrast 2 tests (two-sided) whether, in the partner choice condition, participants who were assigned HH and LL types were more likely to be paired with their own types than participants who were assigned HL and LH types were paired with their own types ($p < .001$). We corrected for multiple testing using a Bonferroni correction.

To examine the stability of pairings, i.e., whether some pairs were more likely to interact for consecutive rounds than others, we fit a multilevel regression model. The dependent variable was the stability of pairings, measured as the length of consecutive interactions between different pairs. The fixed effect was a dummy variable coding if pairings consisted of two similar types (1), or not (0). Same type pairs, such as HH-HH, or HL-HL, were more stable (multilevel model, $t(292) = 12.14$, $b_{\text{similar}} = 2.77$, $p < .001$, 95% CI [2.32, 3.23], Supplementary Table 2).

Supplementary Table 2. Mixed effects regression modelling the number of consecutive rounds that each type interacted with another type.

		Estimate	SE	t	p	95% CI
**Model**	intercept	3.31	0.60	5.48	< 0.001	[2.10, 4.53]
	similar	2.77	0.23	12.14	< 0.001	[2.32, 3.23]

Partner Choice

We fit a multilevel logistic regression model to test how often types were selected as participants' first partner choice. The dependent variable was a dummy variable coding whether a type was ranked first (1), or not (0). The fixed effect consisted of participants' assigned type. Confidence intervals were calculated via the bootstrap method.

Participants who were assigned a high-endowment high-productivity (HH) type were the most preferred partners (i.e., most popular; 65.1% of all first choices of all types consisted of an HH type; multilevel logistic model, $z = 9.76$, $b_{\text{type}} = 5.56$, $p < .001$, 95% CI [4.25, 6.86], Supplementary Table 3). In contrast, participants who were assigned an LL type were the least preferred partner type (i.e., least popular; only 6.7% of all first choices of all types consisted of an LL type; multilevel logistic model, $z = -4.60$, $b_{\text{type}} = -2.40$, $p < .001$, 95% CI [-3.60, -1.20], Supplementary Table 3), although preferences for an LL partner type did not differ significantly from preferences for HL and LH partner types (multilevel logistic model, z = -1.13, $b_{\text{type}} = -0.62$, $p = .775$, 95% CI [-1.87, 0.63], Supplementary Table 3).

Supplementary Table 3. Mixed effects logistic regression modelling how often HH and LL types were selected as participants' first partner choice.

		Estimate	SE	z	p	95% CI
**Model**	intercept	3.09	0.48	6.38	< 0.001	[2.25, 4.10]
	HL type	-5.55	0.68	-8.16	< 0.001	[-7.07, -4.42]
	LH type	-5.15	0.67	-7.73	< 0.001	[-6.57, -3.88]
	LL type	-5.97	0.68	-8.81	< 0.001	[-7.52, -4.61]
**Contrasts**[a]	contrast 1	5.56	0.57	9.76	< 0.001	[4.25, 6.86]
	contrast 2	-2.40	0.52	-4.60	< 0.001	[-3.60, -1.20]
	contrast 3	-0.62	0.54	-1.13	0.776	[-1.87, 0.64]

[a] Contrast 1 tests (two-sided) whether HH types were more often selected as participants' first partner choice than the other types ($p < .001$), and contrast 2 tests (two-sided) whether LL types were less often selected as participants' first partner choice than the other types ($p < .001$). Note that contrast 2 is not orthogonal to contrast 1. We therefore added contrast 3 which tests (two-sided) whether LL types were less often selected as participants' first partner choice than HL and LH types ($p = .776$). We corrected for multiple testing using a Bonferroni correction.

Next, we fit a multilevel logistic regression model to examine how often types were paired with the type of their first choice. The dependent variable was a dummy variable coding whether types could be paired with their first preference (1), or not (0). The fixed effect consisted of participants' assigned type. Confidence intervals were calculated via the bootstrap method.

Modelling results showed that participants who were assigned an HH type were rejected least often, meaning that HH types could be paired with their preferred partner type in most (84%) of the rounds (multilevel logistic model, $z = -11.33$, $b_{type} = -3.49$, $p < .001$, 95% CI [-4.20, -2.79], Supplementary Table 4). Participants assigned an LL type were rejected most often, meaning that they could not be paired with their preferred partner type in most (75%) of the rounds (multilevel logistic model, $z = 6.34$, $b_{type} = 1.85$, $p < .001$, 95% CI [1.18, 2.53], Supplementary Table 4). LL types were also rejected more often than HL and LH types (multilevel logistic model, $z = 2.54$, $b_{type} = 0.78$, $p = .033$, 95% CI [0.08, 1.48], Supplementary Table 4).

Supplementary Table 4. Mixed effects logistic regression modelling how often HH and LL types could be paired with the type of their first choice.

		Estimate	SE	z	p	95% CI
**Model**	intercept	-2.44	0.27	-9.04	< 0.001	[-2.95, -1.94]
	HL type	3.20	0.37	8.74	< 0.001	[2.47, 3.87]
	LH type	3.28	0.37	8.92	< 0.001	[2.53, 4.08]
	LL type	4.01	0.37	10.75	< 0.001	[3.26, 4.75]
**Contrasts**[a]	contrast 1	-3.49	0.31	-11.33	< 0.001	[-4.20, -2.79]
	contrast 2	1.85	0.29	6.34	< 0.001	[1.18, 2.53]
	contrast 3	0.78	0.31	2.54	0.033	[0.08, 1.48]

[a] Contrast 1 tests (two-sided) whether HH types could be paired with the type of their first choice more often than the other types ($p < .001$), and contrast 2 tests (two-sided) whether LL types could be paired with the type of their first choice less often than the other types ($p < .001$). Note that contrast 2 is not orthogonal to contrast 1. We therefore added contrast 3 which tests (two-sided) whether LL types could be paired with the type of their first choice less often than HL and LH types could be paired with their first choice ($p = .033$). We corrected for multiple testing using a Bonferroni correction.

Next, we fit a multilevel logistic regression model to test whether partner types were avoided if paired with them on the previous round. The dependent variable was a dummy variable coding whether, on a given round, participants preferred to be paired with a different partner type (1) or the same partner type (0) compared to the type they preferred to be paired with on the previous round (i.e., ranked as their first choice on the previous round). The fixed effect was participants' previous partner type. For this analysis, the first round was excluded since there was no previous partner. Confidence intervals were calculated via the bootstrap method.

Results showed that participants tried to actively avoid participants who were assigned an LL type after interacting with them (i.e., participants preferred to be paired with a different type after being paired with an LL type; multilevel logistic model, $z = 5.67$, $b_{\text{previous partner}} = 0.72$, $p < .001$, 95% CI [0.43, 1.01], Supplementary Table 5), while they were more likely to prefer a partner assigned an HH type after previously interacting with an HH type (multilevel logistic model, $z = -5.30$, $b_{\text{previous partner}} = -0.86$, $p < .001$, 95% CI [-1.23, -0.49], Supplementary Table 5). Participants were also more likely to switch partner preferences after being paired with an LL type compared to after being paired with an HL and LH type (multilevel logistic model, $z = 3.86$, $b_{\text{previous partner}} = 0.48$, $p < .001$, 95% CI [0.20, 0.77], Supplementary Table 5).

Supplementary Table 5. Mixed effects logistic regression modelling if participants switched partner preferences after being paired with an LL or HH type.

		estimate	SE	z	p	95% CI
**Model**	intercept	-2.58	0.19	-13.54	< 0.001	[-3.01, -2.21]
	previous HL partner	0.66	0.18	3.66	< 0.001	[0.32, 1.02]
	previous LH partner	0.74	0.18	4.10	< 0.001	[0.37, 1.13]
	previous LL partner	1.18	0.19	6.33	< 0.001	[0.78, 1.61]
**Contrasts**[a]	contrast 1	-0.86	0.16	-5.30	< 0.001	[-1.23, -0.49]
	contrast 2	0.72	0.13	5.67	< 0.001	[0.43, 1.01]
	contrast 3	0.48	0.13	3.86	< 0.001	[0.20, 0.77]

[a] Contrast 1 tests (two-sided) whether participants switched partner preferences less often after being paired with an HH type than after being paired with another type ($p < .001$), and contrast 2 tests (two-sided) whether participants switched partner preferences more often after being paired with an LL type than after being paired with another type ($p < .001$). Note that contrast 2 is not orthogonal to contrast 1. We therefore added contrast 3, which tests (two-sided) whether participants switched partner preferences more often after being paired with an LL type than after being paired with HL and LH types ($p < .001$). We corrected for multiple testing using a Bonferroni correction.

Partner Choice Over Time

We fit two multilevel logistic regression models to examine partner choice over time. The first model tested whether preferences for HH partner types decreased over time for participants assigned non-HH types. This model only included data of participants assigned a non-HH type, with the dummy variable coding whether participants preferred HH types (1), or not (0), and the fixed effect being round number. The second model allowed us to test, per type, whether participants increased their preference for their own type over time, with the dependent variable being a dummy variable coding whether participants preferred a partner type identical to their own type (1), or not (0). Fixed effects were participants' own assigned type, round number, and the interaction between participants' own assigned type and round number. In both models, confidence intervals were calculated via the bootstrap method.

Results showed that partner rankings changed over time, with homophily preferences becoming more prevalent in later rounds. In early rounds, most participants, regardless of their own type, preferred to be paired with an HH type. However, the preference for an HH type decreased over time for participants assigned a non-HH type (multilevel logistic model, $z = -9.24$, $b_{\text{round}} = -0.07$, $p < .001$, 95% CI [-0.08, -0.05], Supplementary Table 6). This might be because participants who were assigned a HH type consistently preferred to be paired with each other (multilevel logistic model, $z = 0.15$, $b_{\text{round}} = 0.002$, $p = .879$, 95% CI [-0.03, 0.04], Supplementary Table 7), and were soon unavailable for other types, or because non-HH types wanted to avoid rejection costs. Consequently, participants assigned an HL, LH, and LL type showed an increase in preference for partners of their own type (i.e., homophily; HL: multilevel logistic model, $z = 2.57$, $b_{\text{HL} \times \text{round}} = 0.06$, $p = .010$, 95% CI [0.01, 0.10], Supplementary Table 7; LH: multilevel logistic model, $z =$

1.98, $b_{LH \times round}$ = 0.04, p = .048, 95% CI [0.00, 0.08], Supplementary Table 7; LL: multilevel logistic model, z = 3.19, $b_{LL \times round}$ = 0.07, p = .001, 95% CI [0.02, 0.12], Supplementary Table 7).

Supplementary Table 6. Mixed effects logistic regression modelling HH type partner preferences of non-HH types over time.

	estimate	SE	z	p	95% CI
intercept	1.63	0.29	5.57	< 0.001	[1.09, 2.22]
round	-0.07	0.01	-9.24	< 0.001	[-0.08, -0.05]

Supplementary Table 7. Mixed effects logistic regression modelling preferences for partner types similar to their own type over time.

	estimate	SE	z	p	95% CI
intercept	3.10	0.54	5.78	< 0.001	[2.27, 4.05]
HL type	-6.36	0.76	-8.36	< 0.001	[-7.64, -5.27]
LH type	-5.77	0.74	-7.79	< 0.001	[-6.98, -4.54]
LL type	-7.01	0.76	-9.18	< 0.001	[-8.48, -5.80]
round	0.002	0.02	0.15	0.879	[-0.03, 0.04]
HL type × round	0.06	0.02	2.57	0.010	[0.01, 0.10]
LH type × round	0.04	0.02	1.98	0.048	[0.00, 0.08]
LL type × round	0.07	0.02	3.19	0.001	[0.02, 0.12]

Consequences of Partner Choice on Cooperation and Resource Distribution

Cooperation Between Conditions

All multilevel (logistic) regression models reported with regards to *Cooperation Between Conditions* included random intercepts for participants nested within their group to account for violations of independence, since participants made repeated decisions and were part of a group in which they potentially influenced each other's decisions over time.

We fit a multilevel regression model to examine the impact of condition (partner choice or assigned partners) on cooperation. Participants' relative cooperation rate was the dependent variable, being defined as the average contributions to the public good as a percentage of participant's individual endowment. Fixed effects consisted of condition, round number, and the interaction between condition and round number.

While previous results demonstrated that the partner choice condition segregated the population by type, relative cooperation was higher as a result of partner choice compared to when partners were assigned. Specifically, cooperation decreased over time in the assigned partner condition (multilevel model, $t(7726) = -4.38$, $b_{\text{condition} \times \text{round}} = -0.33$, $p < 0.001$, 95% CI [-0.48, -0.18], Supplementary Table 8), but remained stable across rounds in the partner choice condition (multilevel model, $t(7726) = -1.54$, $b_{\text{round}} = -0.08$, $p = 0.125$, 95% CI [-0.19, 0.02], Supplementary Table 8).

Supplementary Table 8. Mixed effects regression modelling how condition and round number impacted cooperation.

	estimate	SE	t	p	95% CI
intercept	61.95	3.25	19.06	< 0.001	[55.60, 68.31]
condition	-0.12	4.60	-0.03	0.979	[-9.11, 8.87]
round	-0.08	0.05	-1.54	0.125	[-0.19, 0.02]
condition × round	-0.33	0.08	-4.38	< 0.001	[-0.48, -0.18]

We further fit a multilevel logistic regression model to investigate if relative cooperation remained more stable in the partner choice condition, because participants could avoid uncooperative partner types in this condition. The dependent variable was a dummy variable coding whether the partner type, participants preferred to be paired with (i.e., ranked as their first choice), differed from the partner type of their first choice on the previous round (1), or not (0). The fixed effect was a dummy variable coding whether the relative cooperation rate of participants' partner in the previous round was lower than the relative cooperation rate of the participant (1), or not (0). The first round was excluded since there were no previous partner preferences or previous cooperation rates for this round.

Results showed that participants changed their partner preference if, on the previous round, their partner cooperated relatively less than them (multilevel logistic model, $z = 3.67$, $b_{\text{contribution}} = 0.37$, $p < 0.001$, 95% CI [0.17, 0.57], Supplementary Table 9).

Supplementary Table 9. Mixed effects logistic regression modelling the relation between partner preferences in the current round and relative cooperation of one's partner in the previous round.

	estimate	SE	z	p	95% CI
intercept	-2.06	0.14	-14.21	< 0.001	[-2.38, -1.77]
contribution	0.37	0.10	3.67	< 0.001	[0.17, 0.57]

Lastly, we fit a multilevel regression model to investigate if, in addition to an increase in homophily, the decreasing preference for HH types could be driven by participants being assigned a non-HH type avoiding HH type defectors. If so, participants assigned a non-HH type should have met more cooperative non-HH types than cooperative HH types. The model therefore only included participants assigned a non-HH type. The dependent variable was the relative cooperation of one's partner. Fixed effects were a dummy variable coding whether participants were paired with an HH type (1), or not (0), round number, and their interaction.

HH partners cooperated significantly less than non-HH partners when being paired with a non-HH type (multilevel model, $t(2926) = 6.00$, $b_{\text{HHpartner}} = -22.52$, $p < .001$, 95% CI [-29.88, -15.16], Supplementary Table 10), suggesting that the decrease in preference for HH types could (also) be driven by non-HH types avoiding HH type defectors.

Supplementary Table 10. Mixed effects regression modelling if cooperation towards non-HH types is impacted by one's partner type.

	estimate	SE	t	p	95% CI
intercept	61.22	3.13	19.54	< 0.001	[54.98, 67.46]
HH partner	-22.52	3.76	-6.00	< 0.001	[-29.88, -15.16]
round	-0.15	0.08	-1.96	0.051	[-0.31, 0.00]
HH partner × round	0.32	0.28	1.15	0.251	[-0.22, 0.86]

A follow-up analysis was performed to examine whether cooperation depended on the stability of pairings, i.e., the average length of consecutive interactions between pairs. This multilevel regression model included participants' relative cooperation rate as the dependent variable and stability as a fixed effect.

Results showed that participants cooperated relatively more when their pairing was stable (multilevel model, $t(389) = 6.03$, $b_{\text{stability}} = 2.46$, $p < 0.001$, 95% CI [1.67, 3.25], Supplementary Table 11; controlling for type). However, the correlation between stability and cooperation was significantly weaker for participants who were assigned an LL type compared to participants who were assigned an HH type (multilevel model, $t(481) = -2.70$, $b_{\text{stability} \times \text{LL}} = -1.73$, $p = .007$, 95% CI [-2.98, -0.48], Supplementary Table 11).

Supplementary Table 11. Mixed effects regression modelling the relation between stability and cooperation.

	estimate	SE	t	p	95% CI
intercept	41.64	5.13	8.12	< 0.001	[31.69, 51.59]
stability	2.46	0.41	6.03	< 0.001	[1.67, 3.25]
HL type	-8.19	6.20	-1.32	0.188	[-20.25, 3.87]
LH type	22.12	6.22	3.56	< 0.001	[10.05, 34.23]
LL type	9.02	6.19	1.46	0.147	[-3.01, 21.05]
stability × HL type	1.71	1.00	1.71	0.088	[-0.24, 3.67]
stability × LH type	-0.84	0.81	-1.04	0.298	[-2.43, 0.72]
stability × LL type	-1.73	0.64	-2.70	0.007	[-2.98, -0.48]

Cooperation Towards Others

We fit a multilevel regression model to investigate if the low cooperation towards LL types depended on higher value types (i.e., HH, HL, and LH types) reducing their cooperation towards LL types, or not. Therefore, we included LL types as the reference level in the model. The dependent variable was participant's relative cooperation rate. The fixed effects were a dummy variable coding whether participants were paired with an LL type (1) or not (0), participant's own type, and the interaction between these two variables.

Results showed that participants who were assigned a HH, HL, or LH type reduced their cooperation rates significantly when being paired with an LL partner compared to when being paired with a non-LL partner (multilevel model, $t(3899) = -2.18$, $b_{\text{LLpartner} \times \text{HHtype}} = -9.99$, $p = .029$, 95% CI [-18.99, -1.05]; $t(3912) = -6.24$, $b_{\text{LLpartner} \times \text{HLtype}} = -18.51$, $p < .001$, 95% CI [-24.34, -12.72] , $t(3917) = -6.54$, $b_{\text{LLpartner} \times \text{LHtype}} = -18.69$, $p < .001$, 95% CI [-24.29, -13.10], Supplementary Table 12).

Supplementary Table 12: Mixed effects regression modelling how one's own type impacts cooperation rates towards LL types.

	estimate	SE	t	p	95% CI
intercept	52.02	4.61	11.27	< 0.001	[43.03, 61.00]
LL partner type	4.31	1.78	2.42	0.016	[0.82, 7.81]
HH type	15.30	5.72	2.67	0.008	[4.13, 26.47]
HL type	-1.09	5.73	-0.19	0.849	[-12.27, 10.10]
LH type	22.93	5.73	4.00	< 0.001	[11.74, 34.12]
LL partner type × HH type	-9.99	4.58	-2.18	0.029	[-18.99, -1.05]
LL partner type × HL type	-18.51	2.97	-6.24	< 0.001	[-24.34, -12.72]
LL partner type × LH type	-18.69	2.86	-6.54	< 0.001	[-24.29, -13.10]

Next, we fit a multilevel regression model to examine if cooperation depended on whether participants were paired with the type of their first or last choice. Participants' relative cooperation rate was included as the dependent variable, and fixed effects consisted of participants' partner ranking (1st, 2nd, 3rd, or 4th preference), participants' own assigned type, the type of the partner participants were paired with, and round number. Confidence intervals were calculated via the bootstrap method.

While our previous results showed that the partner choice condition prevented the breakdown of cooperation, the results of the current model demonstrate that cooperation levels depended on whether participants were paired with their preferred partner type or not. Participants who were paired with the type of their first choice cooperated relatively more than those who were not paired with their first choice (i.e., were rejected; multilevel model, $z = 13.28$, $b_{\text{ranking}} = 14.11$, $p < 0.001$, 95% CI [11.70, 16.52], Supplementary Table 13; controlling for own type, partner type, and round number). Conversely, participants who were paired with their least preferred partner cooperated relatively less than those not paired with their last choice (multilevel model, $z = -4.98$, $b_{\text{ranking}} = -7.34$, $p < .001$, 95% CI [-10.68, -3.99], Supplementary Table 13; controlling for own type, partner type, and round number). However, participants did not cooperate less with their last (fourth) preference compared to their second and third preference (multilevel model, $z = -1.93$, $b_{\text{ranking}} = -2.96$, $p = .162$, 95% CI [-6.45, 0.53], Supplementary Table 13; controlling for own type, partner type, and round number).

Supplementary Table 13: Mixed effects regression modelling how being paired with participants' first or last choice related to cooperation rates.

		estimate	SE	t	p	95% CI
**Model**	intercept	70.44	4.48	15.74	< 0.001	[61.87, 79.10]
	2nd preference	-10.38	1.20	-8.64	< 0.001	[-12.71, -7.83]
	3rd preference	-15.87	1.34	-11.82	< 0.001	[-18.51, -13.21]
	4th preference	-16.09	1.61	-10.01	< 0.001	[-19.50, -13.09]
	HL type	-12.95	5.38	-2.41	0.017	[-23.23, -2.72]
	LH type	12.66	5.38	2.35	0.020	[2.20, 23.68]
	LL type	-1.93	5.43	-0.36	0.722	[-13.24, 9.37]
	HL partner type	3.41	1.56	2.19	0.029	[0.49, 6.75]
	LH partner type	-2.40	1.56	-1.54	0.123	[-5.48, 0.79]
	LL partner type	-1.72	1.73	-1.00	0.320	[-5.01, 1.75]
	round	-0.11	0.05	-2.18	0.029	[-0.20, -0.01]
		**estimate**	**SE**	**z**	**p**	**95% CI**
**Contrasts**[a]	contrast 1	14.11	1.06	13.28	< 0.001	[11.70, 16.52]
	contrast 2	-7.34	1.47	-4.98	< 0.001	[-10.68, -3.99]
	contrast 3	-2.96	1.54	-1.93	0.162	[-6.45, 0.53]

[a] Contrast 1 tests (two-sided) whether participants cooperated more when being paired with their preferred partner type vs. when they were not paired with this type ($p < .001$), and contrast 2 tests (two-sided) whether participants cooperated less when being paired with the partner type they least preferred vs. when they were not paired with this type ($p < .001$). Note that contrast 2 is not orthogonal to contrast 1. We therefore added contrast 3 which tests (two-sided) whether participants cooperated more when being paired with their least preferred partner type vs. their second and third preference ($p = .162$). We corrected for multiple testing using a Bonferroni correction.

Accumulated Resources

All multilevel regression models reported with regards to *Accumulated Resources* included a random intercept for groups to account for violations of independence, since participants were part of a group in which they potentially influenced each other's decisions. We only included a random intercept for groups, since we analysed aggregated accumulated resources at the end of the public goods game per participant.

To examine whether the accumulated resources per participant (i.e., total number of units owned at the end of the game) depended on condition, participant's assigned type, or the interaction between the two, we fit a multilevel regression model with accumulated resources per participant in the last round as the dependent variable. Fixed effects consisted of participants' own assigned type, condition, and the interaction between participants' own assigned type and condition. Confidence intervals were calculated via the bootstrap method.

We found that accumulated resources did not significantly differ between the partner choice condition and the assigned partner condition, showing that specifically the *distribution* of resources was impacted by the partner choice manipulation (multilevel model, $z = -1.17$, $b_{\text{condition}} = -36.04$, $p = 1.00$, 95% CI [-114.74, 42.67], Supplementary Table 14). Participants who were best off at the beginning of the experiment, those who were assigned an HH type, accumulated more resources than the other types in the partner choice condition (multilevel model, $z = 49.09$, $b_{\text{type}} = 1345.93$, $p < 0.001$, 95% CI [1276.17, 1415.69], Supplementary Table 14). They also accumulated more resources than HH types in the assigned partner condition (multilevel model, $z = 7.70$, $b_{\text{condition}} = 326.95$, $p < 0.001$, 95% CI [218.93, 434.97], Supplementary Table 14). Conversely, participants who were worst off in the beginning of the experiment, those who were assigned an LL type, accumulated less resources than the other types in the partner choice condition (multilevel model,

z = -41.05, b_{type} = -1125.44, $p < 0.001$, 95% CI [-1195.19, -1055.68], Supplementary Table 14), and also compared to LL types in the assigned partner condition (multilevel model, z = -3.34, $b_{condition}$ = -141.71, p = 0.005, 95% CI [-249.73, -33.69], Supplementary Table 14). Finally, LL types also accumulated less resources than HL and LH types in the partner choice condition (multilevel model, z = -26.18, b_{type} = -761.39, $p < .001$, 95% CI [-835.38, -687.40], Supplementary Table 14).

Supplementary Table 14: Mixed effects regression modelling the relationship between accumulated resources, condition, and participant type.

		estimate	SE	t	p	95% CI
**Model**	intercept	2591.90	30.02	86.34	< 0.001	[2533.12, 2650.84]
	HL type	-514.31	33.58	-15.32	< 0.001	[-576.84, -448.50]
	LH type	-1669.95	33.58	-49.73	< 0.001	[-1740.36, -1603.24]
	LL type	-1853.52	33.58	-55.20	< 0.001	[-1921.60, -1785.79]
	assigned partner condition (AP)	-326.95	42.46	-7.70	< 0.001	[-404.32, -246.38]
	HL × AP	307.26	47.49	6.47	< 0.001	[218.66, 399.68]
	LH × AP	387.74	47.49	8.17	< 0.001	[298.00, 496.13]
	LL × AP	468.67	47.49	9.87	< 0.001	[367.77, 561.93]
		**estimate**	**SE**	**z**	**p**	**95% CI**
**Contrasts**[a]	contrast 1	-36.04	30.93	-1.17	1.00	[-114.74, 42.67]
	contrast 2	326.95	42.46	7.70	< 0.001	[218.93, 434.97]
	contrast 3	-141.71	42.46	-3.34	0.005	[-249.73, -33.69]
	contrast 4	1345.93	27.42	49.09	< 0.001	[1276.17, 1415.69]
	contrast 5	-1125.44	27.42	-41.05	< 0.001	[-1195.19, -1055.68]
	contrast 6	-761.39	29.08	-26.18	< 0.001	[-835.38, -687.40]

[a] Contrast 1 tests (two-sided) whether participants accumulated more resources in the partner choice condition than in the assigned partner condition ($p < .001$), contrast 2 tests (two-sided) whether HH types accumulated more resources in the partner choice condition than in the assigned partner condition ($p < .001$), contrast 3 tests (two-sided) whether LL types accumulated less resources in the partner choice condition than in the assigned partner condition ($p = .005$), contrast 4 tests (two-sided) whether HH types accumulated more resources in the partner choice condition than the other types did ($p < .001$), and contrast 5 tests (two-sided) whether LL types accumulated less resources in the partner choice condition than the other types did ($p < .001$). Note that contrast 5 is not orthogonal to contrast 4. We therefore added contrast 6 (two-sided) which tests whether LL types accumulated less resources in the partner choice condition than was accumulated by HL and LH types ($p < .001$). We corrected for multiple testing using a Bonferroni correction.
