# Supplementary material for: Partner choice and cooperation in social dilemmas can increase resource inequality
Source: Nat Commun. 2023 Oct 13;14:6432. doi: 10.1038/s41467-023-42128-2 (PMC10575984; doi:10.1038/s41467-023-42128-2)
Supplement: Supplementary file 3 — Reporting Summary [file 41467_2023_42128_MOESM3_ESM.pdf]

Corresponding author(s): Luuk Snijder

Last updated by author(s): Sep 25, 2023

Reporting Summary

Nature Portfolio wishes to improve the reproducibility of the work that we publish. This form provides structure for consistency and transparency in reporting. For further information on Nature Portfolio policies, see our Editorial Policies and the Editorial Policy Checklist.

Statistics

For all statistical analyses, confirm that the following items are present in the figure legend, table legend, main text, or Methods section.

n/a	Confirmed	
☐	☒	The exact sample size (*n*) for each experimental group/condition, given as a discrete number and unit of measurement
☐	☒	A statement on whether measurements were taken from distinct samples or whether the same sample was measured repeatedly
☐	☒	The statistical test(s) used AND whether they are one- or two-sided<br>*Only common tests should be described solely by name; describe more complex techniques in the Methods section.*
☐	☒	A description of all covariates tested
☐	☒	A description of any assumptions or corrections, such as tests of normality and adjustment for multiple comparisons
☐	☒	A full description of the statistical parameters including central tendency (e.g. means) or other basic estimates (e.g. regression coefficient) AND variation (e.g. standard deviation) or associated estimates of uncertainty (e.g. confidence intervals)
☐	☒	For null hypothesis testing, the test statistic (e.g. *F*, *t*, *r*) with confidence intervals, effect sizes, degrees of freedom and *P* value noted<br>*Give P values as exact values whenever suitable.*
☒	☐	For Bayesian analysis, information on the choice of priors and Markov chain Monte Carlo settings
☒	☐	For hierarchical and complex designs, identification of the appropriate level for tests and full reporting of outcomes
☐	☒	Estimates of effect sizes (e.g. Cohen's *d*, Pearson's *r*), indicating how they were calculated

Our web collection on statistics for biologists contains articles on many of the points above.

Software and code

Policy information about availability of computer code

Data collection	The experiment was programmed in oTree (version 3.4.0), which is Python based (version 3.7.9.). All codes used for programming the experiment and pairing algorithms are publicly available in an OSF repository (https://osf.io/casqz/).
Data analysis	Data was analyzed in R (version 4.0.3). The analysis code for all experiments are also publicly available in the same OSF repository (https://osf.io/casqz/).

For manuscripts utilizing custom algorithms or software that are central to the research but not yet described in published literature, software must be made available to editors and reviewers. We strongly encourage code deposition in a community repository (e.g. GitHub). See the Nature Portfolio guidelines for submitting code & software for further information.

Data

Policy information about availability of data

All manuscripts must include a data availability statement. This statement should provide the following information, where applicable:

- Accession codes, unique identifiers, or web links for publicly available datasets
- A description of any restrictions on data availability
- For clinical datasets or third party data, please ensure that the statement adheres to our policy

The data of our experiment are publicly available in an OSF repository (https://doi.org/10.17605/OSF.IO/CASQZ). There are no restrictions to accessing the data. Additional information can be requested from the corresponding author at l.snijder@fsw.leidenuniv.nl.

Research involving human participants, their data, or biological material

Policy information about studies with human participants or human data. See also policy information about sex, gender (identity/presentation), and sexual orientation and race, ethnicity and racism.

Reporting on sex and gender	69% of our participants was female, 30% of our participants was male (self-reported gender). The gender of 2 participants is missing and 1 participant identified as "other". We did not perform any analyses on gender, since we did not have reason to believe that this would impact our results.
Reporting on race, ethnicity, or other socially relevant groupings	We did not collect data on race or ethnicity.
Population characteristics	Participants were between 18 and 48 years of age (M = 23.81, SD = 4.01).
Recruitment	Participants were recruited via an online recruitment platform from Leiden University (The Netherlands, N = 256, 79% were female, self-reported gender) and via the online platform Prolific (N = 80, 39% were female, self-reported gender). The self-selection of participants to participate in our experiment might have impacted the results. On the one hand, prosocial participants might have been more likely to participate in our experiment. Therefore, we might have overestimated cooperation levels with our current studies. On the other hand, participants, recruited via Leiden University and Prolific, are motivated by earning money, which may have increased individualistic behavior. Therefore, we might have underestimated cooperation levels with our current studies.
Ethics oversight	The experiment was approved by the ethics committee of the Institute of Psychology at Leiden University (2020-11-12-M. Stallen-V2-2726).

Note that full information on the approval of the study protocol must also be provided in the manuscript.

Field-specific reporting

Please select the one below that is the best fit for your research. If you are not sure, read the appropriate sections before making your selection.

☐ Life sciences ☒ Behavioural & social sciences ☐ Ecological, evolutionary & environmental sciences

For a reference copy of the document with all sections, see nature.com/documents/nr-reporting-summary-flat.pdf

Behavioural & social sciences study design

All studies must disclose on these points even when the disclosure is negative.

Study description	We performed a repeated public goods game to investigate how partner choice impacts cooperation under inequality. Individuals differed in ex ante wealth and ability (the value that their cooperation generated). On each round, individuals were either assigned a partner (assigned partner condition), or paired based on their self-indicated preference for a 'partner type' (partner choice condition).<br><br>Data are quantitative. Our key dependent variables are<br>• Partner selection: The frequency by which partner types are selected (over time).<br>• Cooperation: The number of units invested in cooperation on each round.<br>• Accumulated wealth: the number of units that participants earn over time.
Research sample	Participants were students from Leiden University and participants from Prolific. 69% of our participants were female (gender, self-reported), 30% of our participants were male (gender, self-reported) Participants were between 18 and 48 years of age (M = 23.81, SD = 4.01). Participants had to be older than 16 years and speak English fluently. Our sample mostly consists of students, and is therefore not fully representative of the broader population. Our rationale for selecting this sample was based on accessibility, as students are readily available and can easily participate in research studies. To address possible concerns, we are cautious with generalizing results from our sample to the broader population.
Sampling strategy	We collected the data of 336 participants. We used a 2 (condition: partner choice vs assigned partner) x 4 (type: High Endowment – High Productivity factor [HH], High Endowment – Low Productivity factor [HL], Low Endowment – High Productivity factor [LH], Low Endowment – Low Productivity factor [LL]), between-subjects design. Both conditions consisted of 21 groups of eight participants (N = 168 each). Each group consisted of two participants per type, so that there were 42 participants of each type per condition.<br><br>The sample size was determined in advance based on similar research (Hauser, 2019).<br>Hauser, O. P., Hilbe, C., Chatterjee, K. & Nowak, M. A. Social dilemmas among unequals. Nature (2019). doi:10.1038/s41586-019-1488-5
Data collection	The experiment was programmed in oTree (version 3.4.0), which is Python based (version 3.7.9.). Participants were recruited via an online recruitment platform from Leiden University or via the online platform Prolific. Data was collected during the COVID-19 pandemic. Therefore, at the time of the experimental session, participants received a link to the experiment website, so that they

	could participate in this interactive behavioral experiment in real time from home. The experiment (in oTree) was uploaded to a Heroku server such that participants could access the experiment from home.
Timing	Data collection started on 2021-02-01, and finished on 2021-04-19. There was no gap in the collection period.
Data exclusions	No participants were excluded from the final analyses.
Non-participation	No participants dropped out or declined participation. However, their might have been some self-selection of participants to participate in our experiment.
Randomization	Participants were randomly allocated to experimental groups. In the statistical analyses, we included random intercepts for participants nested within their group to account for violations of independence, since participants made repeated decisions and were part of a group in which they potentially influenced each other's decisions over time.

Reporting for specific materials, systems and methods

We require information from authors about some types of materials, experimental systems and methods used in many studies. Here, indicate whether each material, system or method listed is relevant to your study. If you are not sure if a list item applies to your research, read the appropriate section before selecting a response.

Materials & experimental systems

n/a	Involved in the study	
☒	☐	Antibodies
☒	☐	Eukaryotic cell lines
☒	☐	Palaeontology and archaeology
☒	☐	Animals and other organisms
☒	☐	Clinical data
☒	☐	Dual use research of concern
☒	☐	Plants

Methods

n/a	Involved in the study	
☒	☐	ChIP-seq
☒	☐	Flow cytometry
☒	☐	MRI-based neuroimaging
